# Antimicrobial Activity and Brine Shrimp Lethality Bioassay of the Leaves Extract of *Dillenia indica* Linn

**DOI:** 10.4103/0975-1483.62213

**Published:** 2010

**Authors:** AS Apu, MA Muhit, SM Tareq, AH Pathan, ATM Jamaluddin, M Ahmed

**Affiliations:** *Department of Clinical Pharmacy and Pharmacology, Faculty of Pharmacy, University of Dhaka, Dhaka 1000, Bangladesh*

**Keywords:** Antimicrobial activity, *Artemia salina*, brine shrimp lethality bioassay, *Dillenia indica*

## Abstract

The crude methanolic extract of *Dillenia indica* Linn. (Dilleniaceae) leaves has been investigated for the evaluation of antimicrobial and cytotoxic activities. Organic solvent (*n*-hexane, carbon tetrachloride and chloroform) fractions of methanolic extract and methanolic fraction (aqueous) were screened for their antimicrobial activity by disc diffusion method. Besides, the fractions were screened for cytotoxic activity using brine shrimp (*Artemia salina*) lethality bioassay. Among the four fractions tested, *n*-hexane, carbon tetrachloride, and chloroform fractions showed moderate antibacterial and antifungal activity compared to standard antibiotic, kanamycin. The average zone of inhibition was ranged from 6 to 8 mm at a concentration of 400 µg/disc. But the aqueous fraction was found to be insensitive to microbial growth. Compared to vincristine sulfate (with LC_50_ of 0.52 µg/ ml), *n*-hexane and chloroform fractions demonstrated a significant cytotoxic activity (having LC_50_ of 1.94 µg/ml and 2.13 µg/ml, respectively). The LC_50_ values of the carbon tetrachloride and aqueous fraction were 4.46 µg/ml and 5.13 µg/ ml, respectively. The study confirms the moderate antimicrobial and potent cytotoxic activities of *Dillenia indica* leaves extract and therefore demands the isolation of active principles and thorough bioassay.

## INTRODUCTION

The genus *Dillenia* has 60 species, of which *Dillenia indica* Linnaeus (Family: Dilleniaceae) is the most common edible species. Originally from Indonesia, this evergreen tropical tree is now found from India to China. The common names include Chulta (Bengali, Hindi), Bhavya (Sanskrit) and Elephant apple (English). It is a spreading tree and has beautiful white fragrant flowers, toothed leaves, and globose fruits with small brown seeds.[1] The leaf, bark, and fruit of this plant are used as traditional medicine. The juice of *D. indica* leaves, bark, and fruits are mixed and given orally (5-15 ml, two to five times daily) in the treatment of cancer and diarrhea.[2] The fruit juice of this plant has cardiotonic effect, used as cooling beverage in fever and also employed in cough mixture.[3] The solvent extracts of fruits of *D. indica* are reported to have antioxidant activity.[4] CNS depressant activities in mice were found from the alcoholic extract of the leaves of *D. indica*.[5] Considering the traditional uses of *D. indica* plant parts, leaves can be the source of many modern medicines.

A survey of the published literature shows that there are a number of different methods used for the assessment of antimicrobial activity; however, there is no one method that is used by all researchers and no inclusive study to determine which one is the best method for *in vitro* assay.[6] Majority of the researchers uses one of the three following methods for the assessment of antimicrobial activity: Disc diffusion, agar dilution, and broth dilution/microdilution method. The disc diffusion method (also known the zone of inhibition method)[7] is probably the most widely used of all methods used for testing antibacterial and antifungal activity.[6] It requires only small amounts of the test substance (10-30 µl), can be completed by research staff with minimal training, and as such may be useful in field situations.[6] Several researchers have used the method to identify the antibacterial and antifungal activities of the plant extracts,[8] compounds isolated from plants,[9] and also to find out the antimicrobial resistant strains of microorganisms.[10,11] It is important to note that the disc diffusion method demonstrated activity *in vitro* does not always translate to activity *in vivo*.[6]

The brine shrimp lethality bioassay is rapid (24 h), simple (e.g., no aseptic techniques are required), easily mastered, inexpensive, and requires small amounts of test material (2-20 mg or less).[12] The bioassay has a good correlation with cytotoxic activity in some human solid tumors and with pesticidal activity.[12,13] This test was proposed by Michael *et al*.[14] and modified by others.[15,16] Since its introduction, this *in vivo* lethality test has been successively employed for providing a frontline screen that can be backed up by more specific and more sophisticated bioassays once the active compounds have been isolated.

The objective of this research work was to investigate the antimicrobial and cytotoxic activities of the different solvent fractions of crude methanolic extract of *D. indica* leaves.

## MATERIALS AND METHODS

### Collection of plant material

The plant sample of *D. indica* was collected from Rangpur, Bangladesh, in the month of March 2007. The plant was identified and a voucher specimen (Accession number DACB 34359) representing this collection has been deposited in the Bangladesh National Herbarium, Dhaka, for further reference.

### Preparation, extraction and fractionation of plant material

The freshly separated leaves of the plant were cut into small pieces, sun dried, and subsequently dried in the oven for 24 h at low temperature to grind these into coarse powder (40-mesh).

About 500 g of powdered leaves was taken in a 5 l round bottom flask and soaked in 2 l of methanol. The container with its content was sealed with cotton plug and aluminum foil and kept at room temperature for a period of 3 days accompanying occasional shaking and stirring. The extract was filtered through fresh cotton plug followed by Whatman No.1 filter paper. The filtrate was then concentrated and dried by a rotary evaporator (Heidolph, UK) at low temperature (39°C). The weight of the crude extract thus obtained from leaves was 7 g.

Solvent-solvent fractionation of the crude methanolic extract was conducted by using the protocol designed by Kupchan[17] and modified by Wagenen *et al*.[18] 5 g of the obtained methanolic crude extract was triturated with 90% methanol. The prepared solution was then fractionated successively using solvents of increasing polarity, such as, *n*-hexane (HX), carbon tetrachloride (CT), and chloroform (CF). The aqueous methanolic fraction was preserved as aqueous fraction (AQ). All the four fractions were evaporated to dryness by using rotary evaporator and then kept in beakers for further analysis (HX 820 mg, CT 550 mg, CF 665 mg and AQ 400 mg).

### Antimicrobial screening

Antibacterial and antifungal activities of crude extracts were tested by the paper disc diffusion method.[7] Thirteen bacterial strains, which included 5 gram-positive and 8 gram-negative organisms, and 3 fungi collected from the Institute of Nutrition and Food Science (INFS), University of Dhaka, Bangladesh, as pure cultures were used. Microorganisms were maintained on the nutrient agar medium (Merck, Germany).

The sterile Matricel (BBL, cocksville USA) 6.0 mm filter paper discs were impregnated with 400 µg of each of the sterile test substances and dried to evaporate the residual solvent (methanol). Standard kanamycin discs (30 µg/ disc) were used as positive control to ensure the activity of standard antibiotic against the test organisms. The sample discs, the standard antibiotic discs, and dried blank disc impregnated with methanol (negative control) were placed gently on the previously marked zones in the agar plates pre-inoculated with the test bacteria and fungi. The plates were then kept in a refrigerator at 4°C for about 24 h upside down to allow sufficient diffusion of the materials from the discs to the surrounding agar medium. The plates were then inverted and kept in an incubator at 37°C for 24 h.

The antimicrobial activity of the test agents were measured by their activity to prevent the growth of the microorganisms surrounding the discs which gave clear, distinct zone of inhibition. The antimicrobial activity of the test agents was determined by measuring the diameter of zone of inhibition expressed in mm.[6]

### Brine shrimp lethality bioassay

**Figure 1:**
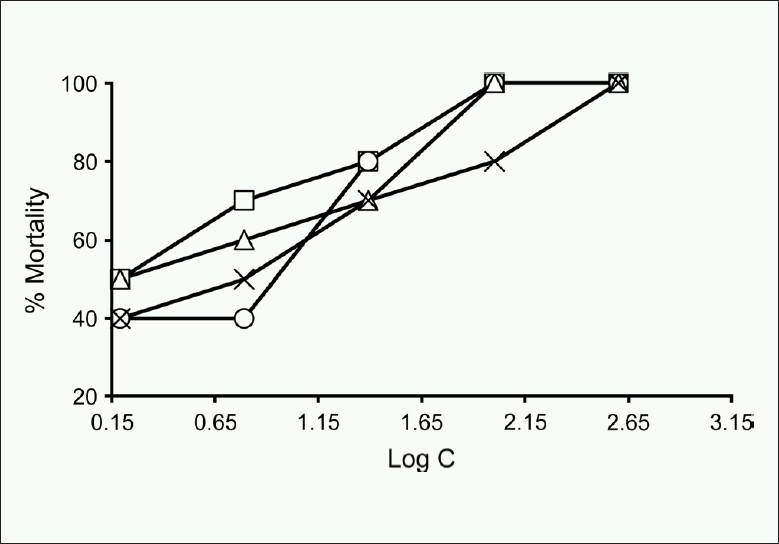
Plot of log concentration of *n*-hexane (— □ —), carbon tetrachloride (— ○ —), chloroform (— ∆ —) and aqueous (— × —) fraction of methanolic extract versus percent shrimp mortality after 24 h of exposure

The brine shrimp lethality bioassay was used to predict the cytotoxic activity[15,19] of the *n*-hexane, carbon tetrachloride, chloroform, and aqueous fractions from methanolic crude extracts. For the experiment, 4 mg of each of the extracts was dissolved in dimethylsulfoxide (DMSO) and solutions of varying concentrations (400, 200, 100, 50, 25, 12.5, 6.25, 3.13, 1.56, 0.78 µg/ ml) were obtained by the serial dilution technique using simulated seawater. The solutions were then added to the pre-marked vials containing 10 live brine shrimp nauplii in 5 ml simulated seawater. After 24 h, the vials were inspected using a magnifying glass and the number of survived nauplii in each vial was counted. The mortality endpoint of this bioassay was defined as the absence of controlled forward motion during 30 s of observation.[20] From this data, the percent of lethality of the brine shrimp nauplii for each concentration and control was calculated. An approximate linear correlation was observed when logarithm of concentration versus percentage of mortality[21] was plotted on the graph paper and the values of LC_50_ were calculated using Microsoft Excel 2003 [Figure 1]. Vincristine sulphate was used as positive control.

## RESULT AND DISCUSSION

With the exception of aqueous fraction, all the other fractions of *D. indica* leaves were active against most of the tested organisms [Table 1]. The average zone of inhibition produced by the *n*-hexane, carbon tetrachloride, and chloroform fraction was ranged from 6-8 mm, 7-8 mm, and 6-7 mm, respectively, at a concentration of 400 µg/ disc. Against the *Escherichia coli*, only chloroform fraction was active (zone of inhibition was 7 mm) and carbon tetrachloride fraction exhibited highest antimicrobial activity compared to other solvent fractions. In both the cases of bacteria and fungi, the zone of inhibition was found to be 6-8 mm.

**Table 1:** Antimicrobial activity of chloroform, carbon tetrachloride, *n*-hexane, and aqueous fraction of methanolic extract of *Dillenia indica* leaves and positive control kanamycin

Test microorganisms	Diameter of zone of inhibition (mm ± SD) (*n* = 3)				
	CF	CT	HX	AQ	KM
Gram-positive bacteria					
*Bacillus cereus*	7 ± 0.3	8 ± 0.2	7 ± 0.3	-	36 ± 0.2
*Bacillus megaterium*	7 ± 0.2	8 ± 0.4	7 ± 0.1	-	37 ± 0.3
*Bacillus subtilis*	7 ± 0.3	8 ± 0.2	6 ± 0.3	-	40 ± 0.3
*Staphylococcus aureus*	-	8 ± 0.3	7 ± 0.2	-	28 ± 0.2
*Sarcina lutea*	7 ± 0.2	8 ± 0.3	7 ± 0.3	-	31 ± 0.5
Gram-negative bacteria					
*Escherichia coli*	7 ± 0.2	-	-	-	32 ± 0.5
*Pseudomonus aeruginosa*	6 ± 0.5	7 ± 0.2	7 ± 0.3	-	30 ± 0.4
*Salmonella paratyphi*	7 ± 0.4	8 ± 0.3	7 ± 0.1	-	33 ± 0.3
*Salmonella typhi*	7 ± 0.1	8 ± 0.3	7 ± 0.5	-	35 ± 0.1
*Shigella boydii*	7 ± 0.3	8 ± 0.1	6 ± 0.4	-	31 ± 0.5
*Shigella dysenteriae*	6 ± 0.3	7 ± 0.3	7 ± 0.2	-	35 ± 0.3
*Vibrio mimicus*	7 ± 0.2	8 ± 0.4	8 ± 0.1	-	34 ± 0.2
*Vibrio parahemolyticus*	7 ± 0.3	8 ± 0.3	8 ± 0.5	-	35 ± 0.3
Fungi					
*Candida albicans*	7 ± 0.3	8 ± 0.3	7 ± 0.4	-	32 ± 0.3
*Aspergillus niger*	7 ± 0.1	8 ± 0.3	7 ± 0.1	-	34 ± 0.1
*Sachoromyces cerevacae*	6 ± 0.4	8 ± 0.2	7 ± 0.3	-	31 ± 0.3

“−” = Indicates no zone of inhibition

The LC_50_ values obtained from brine shrimp lethality bioassay [Tables 2 and 3] were 1.94, 4.46, 2.13, and 5.13 µg/ml for *n*-hexane (HX), carbon tetrachloride (CT), chloroform (CF), and aqueous (AQ) fraction, respectively. Compared to positive control (vincristine sulphate, VS, LC_50_ 0.52 µg/ml), all the fractions tested showed good brine shrimp larvicidal activity. Again the crude extracts resulting in LC_50_ values less than 250 µg/ml were considered significantly active and had the potential for further investigation.[22] The cytotoxic activity exhibited by the solvent fractions was promising and this clearly indicates the presence of potent bioactive compounds.[15]

**Table 2:** Effect of *n*-hexane, carbon tetrachloride, chloroform and aqueous fraction of methanolic extract and positive control vincristine sulphate on brine shrimp

Conc. (µg/ml)	Log C	% mortality				LC_50_ (µg/ml)				Vincristine sulphate			
		HX	CT	CF	AQ	HX	CT	CF	AQ	Conc. (µg/ml)	Log C	% mortality	LC_50_ (µg/ml)
400	2.602	100	100	100	100	1.94	4.46	2.13	5.13	40	1.602	100	0.52
200	2.301	100	100	100	100					20	1.301	100	
100	2.000	100	100	100	80					10	1.000	100	
50	1.698	90	80	80	70					5	0.699	90	
25	1.398	80	80	70	70					2.50	0.398	80	
12.5	1.097	80	60	70	60					1.25	0.097	60	
6.25	0.796	70	50	60	50					0.63	−0.201	50	
3.13	0.495	50	40	50	40					0.31	−0.509	40	
1.56	0.194	50	40	50	40					0.16	−0.796	30	
0.78	−0.108	30	30	40	30					0.078	−1.108	20	

**Table 3:** The result of cytotoxic activity of *n*-hexane (HX), carbon tetrachloride (CT), chloroform (CF), and aqueous (AQ) fraction of methanolic extract and positive control vincristine sulphate (VS) on brine shrimp

Sample	LC_50_ (µg/ml)	Regression equation	R^2^
VS	0.52	y = 33.256x+ 58.740	0.9580
HX	1.94	y = 25.972x+ 42.602	0.9253
CT	4.46	y = 29.192x+ 30.585	0.8837
CF	2.13	y = 24.159x+ 41.863	0.9569
AQ	5.13	y = 26.575x+ 30.849	0.9706

## CONCLUSION

The antimicrobial and cytotoxic activities of various fractions of *D. indica* leaves, found in this study, may explain some of the traditional medicinal uses of this plant. These could be of particular interest in relation to find out its unexplored efficacy and can be a potential source of chemically interesting and biologically important drug candidates.
